# Maternal type 1 diabetes, preterm birth, and risk of intellectual disability in the offspring: A nation-wide study in Sweden

**DOI:** 10.1192/j.eurpsy.2024.4

**Published:** 2024-01-22

**Authors:** Martina Persson, Kristina Tedroff, Weiyao Yin, Mikael Andersson Franko, Sven Sandin

**Affiliations:** 1 Department of Clinical Science and Education, Division of Pediatrics, Karolinska Institutet, Stockholm, Sweden; 2 Sachsska Children’s and Youth Hospital, Stockholm, Sweden; 3 Neuropediatric Unit, Department of Women’s and Children’s Health, Karolinska Institutet, Stockholm, Sweden; 4 Karolinska University Hospital, Stockholm, Sweden; 5 Department of Medical Epidemiology and Biostatistics, Karolinska Institutet, Stockholm, Sweden; 6 Department of Psychiatry, Icahn School of Medicine at Mount Sinai, New York, NY, USA; 7 Seaver Autism Center for Research and Treatment at Mount Sinai, New York, NY, USA

**Keywords:** Type 1 diabetes, Intellectual disability, Population-based, Preterm birth, neurdevelopment

## Abstract

**Objective:**

There are few data on long-term neurological or cognitive outcomes in the offspring of mothers with type 1 diabetes (T1D). The aims of this study were to examine if maternal T1D increases the risk of intellectual disability (ID) in the offspring, estimate the amount of mediation through preterm birth, and examine if the association was modified by maternal glycated hemoglobin (HbA1c).

**Design:**

Population-based cohort study using population-based data from several national registries in Sweden.

**Setting and participants:**

All offspring born alive in Sweden between the years 1998 and 2015.

**Main outcome measure:**

The risk of ID was estimated through hazard ratios with 95% confidence intervals (HR, 95% CI) from Cox proportional hazard models, adjusting for potential confounding. Risks were also assessed in mediation analyses and in subgroups of term/preterm births, in relation to maternal HbA1c and by severity of ID.

**Results:**

In total, 1,406,441 offspring were included. In this cohort, 7,794 (0.57%) offspring were born to mothers with T1D. The risk of ID was increased in offspring of mothers with T1D (HR; 1.77, 1.43–2.20), of which 47% (95% CI: 34–100) was mediated through preterm birth. The HRs were not modified by HbA1c.

**Conclusion:**

T1D in pregnancy is associated with moderately increased risks of ID in the offspring. The risk is largely mediated by preterm birth, in particular for moderate/severe cases of ID. There was no support for risk-modification by maternal HbA1c.

## Introduction

Maternal type 1 diabetes (T1D) increases the risks of major malformations in the central nervous system and acute neurological morbidity in the offspring [1–3]. Also, the high rates of preterm birth in T1D may adversely impact the developing nervous system. However, there are few data on long-term neurological outcomes in the offspring of mothers with T1D.

Intellectual disability (ID) is a childhood neurodevelopmental disorder characterized by intellectual as well as adaptive deficits [4]. ID is more common in boys than in girls, and the worldwide prevalence of ID is estimated to 1–3% [5, 6]. Approximately one-third of ID cases are due to genetic factors [7], but a number of environmental exposures have also been associated with increased risk [8]. However, the underlying cause of ID often remains unknown [7].

Results from recent meta-analyses support an overall association between maternal preexisting diabetes and cognitive disorders in the offspring [9, 10]. However, the risks of ID were not assessed. Also, as preterm birth is a well-known risk factor for neurological developmental disorders [8] and affecting around one in four pregnancies with T1D [11], the potential mediating role of preterm birth should be taken into account when assessing risks.

The aim of this study was to examine if maternal T1D increases the risk of offspring ID, estimate the amount of mediation through preterm birth, and examine if the association was modified by maternal glycemic control, measured as HbA1c.

## Methods

### Study population

This prospective, population-based study used information from the Swedish Medical Birth Register (MBR) and included all children born alive in Sweden between 1998 and 2015. The MBR records data on all pregnancies and deliveries in Sweden since 1973, with information on demography, maternal, and infant diagnoses [12]. Diagnoses are coded according to the International Classification of Diseases (ICD-10, for specific codes, see Table 1) and assigned by clinical specialists. Individual-level information from the Swedish national registries were linked using the personal identification number assigned to all mothers and their offspring [13]. To reduce confounding by maternal origin, we only included offspring of mothers born in the Nordic countries. Multiple births increase the risk of preterm delivery and fetal growth restriction [14], both of which may increase the risk of ID [6–8]. Also, the risks of ID are higher in males than in females [5]. Accordingly, all main analyses were repeated in singleton births only and stratified by fetal sex.

**Table 1.** Cohort description

Covariate	Mothers without T1D Number of children (%)	Mothers with T1D Number of children (%)
Children (%male)	1,398,441 (51.5%)	8,000 (51.0%)
Intellectual disability (ID)	8,439 (0.6%)	84 (1.1%)
Degree of ID		
Mild	5175 (61.3%)	54 (64.3%)
Moderate	1102 (13.1%)	8 (9.5%)
Severe	573 (6.8%)	4 (4.8%)
Other	1589 (18.8%)	18 (21.4%)
Pre terms birth (<37 weeks gestation)	80,014 (5.7%)	1,858 (23.2%)
Birth year		
1998–2003	432,362 (30.9%)	2,025 (25.3%)
2004–2009	479,926 (34.3%)	2,772 (34.6%)
2010–2015	486,153 (34.8%)	3,203 (40.0%)
Mothers age at delivery		
<20	11,887 (0.9%)	73 (0.9%)
20–29	536,331 (38.4%)	3,163 (39.5%)
30–39	789,711 (56.5%)	4,403 (55.0%)
≥40	60,512 (4.3%)	361 (4.5%)
Fathers age at delivery		
<20	3,849 (0.3%)	18 (0.2%)
20–29	357,337 (25.6%)	2,077 (26.0%)
30–39	854,991 (61.1%)	4,831 (60.4%)
≥40	182,264 (13.0%)	1,074 (13.4%)
Size for gestational age		
SGA	25,789 (1.9%)	92 (1.2%)
AGA	1,278,108 (94.3%)	4,771 (61.4%)
LGA	50,863 (3.8%)	2,905 (37.4%)
Maternal psychiatric history	130,798 (9.4%)	1,346 (16.8%)
Paternal psychiatric history	83,052 (5.9%)	587 (7.3%)
Mother’s BMI (Q1/Median/Q3)*	23.5 (21.5–26.5)	25.1 (22.8–28.3)
Underweight#	26,903 (2.1%)	35 (0.5%)
Normal weight#	785,29o (62.2%)	3,536 (49.0%)
Overweight#	305,528 (24.2%)	2,410 (33.4%)
Obese#	144,006 (11.4%)	1,235 (17.1%)
Maternal education		
Primary	113,432 (8.1%)	784 (9.8%)
Secondary	614,360 (43.9%)	3,713 (46.4%)
University	670,649 (48.0%)	3,503 (43.8%)
Paternal education		
Primary	144,570 (10.3%)	891 (11.1%)
Secondary	714,943 (51.1%)	4,405 (55.1%)
University	538,928 (38.5%)	2,704 (33.8%)

Abbreviations: AGA, appropriate for gestational age; LGA, large for gestational age; SGA, small for gestational age; # underweight, BMI < 18.5; normal weight, BMI 18.5–25; overweight, BMI 25–30; obese, BMI ≥ 30; * Q1, first quartile (25th percentile); Q3, third quartile (75th percentile).

### Outcomes

The primary outcome was a diagnosis of ID in the offspring. Secondary outcomes included subgroups of ID by severity, that is, mild, moderate, severe, and profound ID, available in the ICD coding system. This was added since severe or profound ID most often coexists with other conditions, such as cerebral palsy or epilepsy that are more common after preterm birth while mild ID could be identified in otherwise healthy individuals. As part of the Swedish Child-Health Care Program, all neonates and children are regularly assessed with respect to growth and psychomotor development. A more detailed assessment of motor skills, language development, cognitive, and social performance is done at 2.5 and 4 years of age. In case of suspected ID or other neurodevelopmental disorder, the child is referred for evaluation by a team of specialists of child psychologists, pediatricians or child psychiatrists, and speech and language therapists. Potential diagnoses of ID are assigned by the clinical specialists and classified, since 1997 according the 10th version of ICD (ICD 10) and registered in the Swedish National Patient Register (NPR) [15]. The NPR includes all inpatient diagnoses in Sweden since 1973 and outpatient visits from 2001, with almost complete national coverage since 2005. Data in the NPR is considered of high validity [15]. Besides data on potential ID diagnoses in the offspring, we also collected information from the NPR on psychiatric diagnoses in the parents. In this study, participants were censored at the first diagnosis of ID. However, in cases where the first diagnosis of ID was “unspecified,” and the same patient later received a specified ID diagnosis stating the level of ID, the latter was registered while keeping the date of the first received diagnosis. Information on diagnostic codes is available in Supplementary Table S1.

### Exposure

We identified women with T1D using the ICD-10 code E10 recorded in the MBR or in the NPR. The diagnosis of T1D is based on recommendations by the International Society for Pediatric and Adolescent Diabetes [16] and the American Diabetes Association [17]. In Sweden, women with T1D are cared for at specialist clinics for Diabetes and Endocrinology. As soon as pregnancy is recognized, women are referred to specialist antenatal care. Based on data in the Swedish National Diabetes Register (NDR) [18], the mean of each individual’s HbA1c values recorded within 1 year before and until 90 days after conception was calculated.

### Covariates

We considered several potential confounders and mediators. The year of birth was included to adjust for a possible confounding time trend in ID incidence. Parental age may impact the risk of preterm birth [19, 20] and ID [8, 21] and may thus introduce confounding. Data on maternal age were retrieved from the MBR and paternal age from the Swedish Multigenerational Register [22]. Gestational age (GA) and fetal size may also impact the risk of ID [6]. Estimated GA in weeks at delivery was collected from the MBR and is based on information from an early second-trimester ultrasound scan or, in less than 1% of pregnancies, using the date of the last menstrual period. Offspring were categorized as preterm (i.e., birth <37 completed weeks) and term (i.e., birth 37–42 weeks) and according to size at birth as small for GA and sex (birth weight <10th percentile), appropriate (AGA, birth weight 10–90th percentiles), and large for GA and sex (birth weight >90th percentile), based on Swedish reference data on fetal growth [23]. Maternal BMI at the first antenatal visit was calculated based on self-reported height and measured weight and categorized as underweight: BMI <18.5 kg/m^2^, normal weight: BMI 18.5–24.9, overweight BMI: 25–29.9 and obesity BMI: ≥30 [24]. Information on parental history of psychiatric morbidity at the date of delivery, that is, a potential diagnosis of any psychiatric disease and including neuropsychiatric diagnoses (autism spectrum disorder, ADD, ADHD, and ID) was obtained from the NPR [15] (for diagnostic codes please see Supplementary Table S1). Maternal and paternal socioeconomic status were assessed as the maximum attained level of education at the date of delivery, based on information from Statistics Sweden [25] and categorized as in Table 1.

### Statistical methods

Absolute numbers, proportions, and incidence rates of ID were calculated for offspring of mothers with and without T1D. Kaplan–Meier survival curves of age-cumulative probability of ID in offspring of mothers with T1D were calculated. We estimated the relative risks of ID with hazard ratios (HR) and two-sided 95% likelihood confidence intervals (CI) calculated from Cox proportional hazard regression models. Each child was followed from 1 year of age and until the first diagnosis of ID, emigration, death, or end of follow-up on December 31, 2017. A “crude model” estimated the risk of ID adjusted for birth year by natural cubic splines with two knots at the first and second tertiles. Next, risk estimates were additionally adjusted for potential confounders; maternal and paternal age by natural cubic splines, each with two knots at the first and second tertiles, parental psychiatric history (yes/no), and parental level of education (Primary School, Secondary School, University) at the date of delivery. This model was also repeated in singletons. To examine potential time trends in the relative risk of ID, the study was divided in two 5-year periods and adjusted relative risks of ID in children, all with 10 years of follow-up, were calculated for study periods 1 and 2 separately. To evaluate potential unmeasured confounding, we calculated *E*-values for the direct and mediated effects [25].

#### The role of preterm birth

The role of preterm birth as a mediator of ID risk was assessed in a series of analyses. First, the main analyses were repeated stratified by preterm birth. Next, a formal mediation analysis using logistic regression for the event of ID before 10 years of age was performed, assessing the mediating role of preterm birth for risk of ID [26]. Crude and adjusted models were fitted.

#### Supplementary analyses

We performed a series of complementary analyses: (1) risk of ID is higher in boys, and analyses were stratified by sex. First, we fitted a crude model adjusted for birth year and then also for parental age, education, and psychiatric history; (2) sex-specific HR’s were also calculated stratified by preterm birth and by ID severity; (3) maternal overweight and obesity is common and increase the risk of complications; therefore, we estimated the risks of ID stratified by maternal overweight and obesity in pregnancies with and without T1D; (4) as fetal size at birth impact the risk of ID, HR’s of ID were calculated stratified by fetal size and sex; (5) we examined the association between maternal HbA1c and offspring ID among mothers with T1D using Cox regression and by calculating quintiles of HbA1c as main exposure and adjusting for potential confounding by birth year and parental age by natural splines, parental psychiatric history, and education; (6) since T1D duration is closely linked to maternal age [27, 28] and T1D may affect biological age [29], we estimated the risk of offspring ID as a function of maternal age; and (7) the main analyses were repeated in children with at least 10 years’ follow-up.

All tests of statistical hypotheses were two-sided with 5% level of significance. The main hypotheses are based on a sequence of three statistical tests (adjusted overall T1D; mediation direct and indirect) which follows a closed-procedure [30] with an overall familywise 5% level of significance. All analyses were performed using the statistical package R, version 3.6.1. We examined the assumption of proportional hazards by visual inspection of weighted Schoenfeld residuals and score tests. No data were imputed. The study was approved by the Regional Ethics committee in Stockholm, id no: 2017/1875-31/2.

## Results

Our study cohort included 1,425,788 offspring born in Sweden in 1998–2015. Then, 4,968 individuals were excluded due to death, emigration, or with only one ID diagnosis before 1 year of age. Data on any covariate were missing for 14,379 individuals. Of the 1,406,441 individuals in the dataset, 8,000 offspring were born to mothers with T1D. In total, 84 (1.05%) of offspring to mothers with T1D had a diagnosis of ID and 8,439 (0.60%) of offspring to mothers without T1D. 23.5% of pregnancies with T1D ended preterm, compared to 5.7% in pregnancies without diabetes (Table 1).

The cumulative incidence of ID in offspring of mothers with T1D is illustrated in Supplementary Figure S1. Comparing offspring of mothers with and without T1D, the crude HR of ID was estimated to be 1.90 (CI: 1.53–2.36). The adjusted HR was estimated at HR 1.71 (CI: 1.38–2.11) (Table 2).

**Table 2.** Relative risk (hazard ratios) of ID in offspring to mothers with T1D, compared to offspring to mothers without T1D

Model	Number of subjects	Rate (cases; person years)	Hazard ratio (95% CI)
Crude#	1,406,441	63 (8,523; 13,553,218)	1.90 (1.53–2.36)
Adjusted##			1.71 (1.38–2.11)
Repeated main analysis by subgroups			
Singletons###	1,365,316	62 (8,157; 13,147,887)	1.77 (1.43–2.20)
Children born 1998–2002 ####	356,379	62 (2,194; 3,526,123)	1.64 (1.03–2.60)
Children born 2003–2007 ####	397,230	58 (2,250; 3,892,116)	1.69 (1.13–2.53)

Abbreviation: CI, two-sided 95% confidence interval.

*Note:* # Crude: Adjusting for birth years by natural cubic splines; ##Adjusted: Crude + further adjusted for maternal and paternal age by natural cubic splines, parental psychiatric history (yes/no) and maternal and paternal education at delivery; ### Adjusted model excluding twins, that is, singletons only; ####Adjusted model – Each child followed for at least 10 years to allow a comparison not affected by differences in length of follow-up.

### Mediation by preterm birth

In subgroup analyses, risks of ID were statistically significantly increased in children born at term, in both crude and adjusted models, but not in preterm born (Table 3). The mediation analysis estimated the total adjusted risk of ID at RR = 1.50 (CI: 1.20–1.86), with a direct effect by maternal T1D of RR = 1.26 (CI: 1.01–1.57) and mediated by preterm birth estimated at RR = 1.19 (CI: 1.17–1.21), corresponding to 47% (CI: 33–95) of the total effect. The *E*-values necessary to refute the HR for direct effect of T1D and for the mediating effect by preterm birth are shown in Table 4. The mediating effect by preterm birth was comparable between sexes (Table 4). The proportion of mediation by preterm birth was estimated to be 38% (CI: 26–89) for mild and at >60% for moderate/severe ID (Table 4). Risks of mild ID were statistically significantly increased in offspring of mothers with T1D also after taking preterm birth into account. However, the risks of moderate/severe ID were not increased (Table 5).

**Table 3.** Hazard ratios for ID in subgroups of term and preterm births

Model	Number of subjects	Rate (cases; person years)	Hazard ratio (95% CI)
	Preterm born children		
Crude#	81,872	165 (1,312; 796,974)	1.06 (0.74–1.54)
Adjusted##			0.96 (0.66–1.39)
*Subgroup:*	64,126	177 (1,098; 621,614)	0.96 (0.66–1.38)
Singletons###			
	Term born children		
Crude#	1,324,569	57 (7,211; 12,756,245)	1.81 (1.39–2.36)
Adjusted##			1.66 (1.27–2.17)
*Subgroup:*	1,301,190	56 (7,059; 12,526,273)	1.69 (1.30–2.20)
Singletons###			

*Note:* # Crude: Adjusting for birth years by natural cubic splines; ## Adjusted for birth year, parental age by natural splines, parental psychiatric history and education; CI, two-sided 95% confidence interval; ### Adjusted model in subset including singletons only; Rate: cases per 100,000 person years.

**Table 4.** Mediation of effect from maternal T1D to offspring ID, mediated by preterm birth. Mediation analyses assessing risk by relative risks (RR) from log-binomial regression T1D → ID and for preterm → ID

A: Any ID (mild, moderate or severe)								
Model	Number of subjects	ID (%)	Direct effect^£^ RR (95% CI)	*E*-value	Mediation effect^£^ RR (95% CI)	*E*-value	Proportion mediation^£^ % (95% CI)	Total effect RR (95% CI)
Crude#	1,406,441	8,523 (0.61%)	1.45 (1.14–1.76)	2.26 (1.54–2.92)	1.30 (1.27–1.33)	1.92 (1.86–1.99)	49 (40–71)	1.88 (1.48–2.31)
Adjusted##	1,406,441	8,523 (0.61%)	1.26 (1.01–1.57)	1.83 (1.11–2.52)	1.19 (1.17–1.21)	1.66 (1.62–1.71)	47 (33–95)	1.50 (1.20–1.86)
Subgroups:								
Singletons##	1,365,316	8,157 (0.60%)	1.26 (0.98–1.54)	1.83 (1.00–2.45)	1.20 (1.18–1.23)	1.70 (1.64–1.76)	49 (36–102)	1.52 (1.19–1.85)
Males##	723,661	5,172 (0.71%)	1.12 (0.82–1.46)	1.49 (1.00–2.28)	1.19 (1.17–1.22)	1.67 (1.62–1.74)	65 (31–526)	1.33 (0.96–1.74)
Females##	682,780	3,351 (0.49%)	1.50 (1.07–2.00)	2.37 (1.34–3.41)	1.18 (1.15–1.21)	1.64 (1.57–1.71)	35 (24–75)	1.76 (1.27–2.36)

B: Mild ID								
Model	Number of subjects	Mild ID (%)	Direct effect^£^ RR (95% CI)	*E*-value (95% CI)	Mediation effect^£^ RR (95% CI)	*E*-value (95% CI)	Proportion mediation^£^ % (95% CI)	Total effect RR (95% CI)
Crude#	1,406,441	5,229 (0.37%)	1.60 (1.18–2.04)	2.58 (1.64–3.50)	1.26 (1.23–1.30)	1.83 (1.76–1.92)	41 (33–63)	2.02 (1.51–2.58)
Adjusted##	1,406,441	5,229 (0.37%)	1.34 (1.01–1.71)	2.01 (1.11–2.81)	1.16 (1.13–1.18)	1.58 (1.51–1.64)	38 (26–89)	1.55 (1.18–1.98)
Subgroups								
Singletons##	1,365,316	4,988 (0.37%)	1.36 (1.02–1.73)	2.07 (1.16–2.85)	1.16 (1.14–1.19)	1.60 (1.54–1.67)	38 (27–88)	1.59 (1.19–2.02)
Males##	723,661	3,197 (0.44%)	1.27 (0.89–1.73)	1.85 (1.00–2.85)	1.17 (1.14–1.20)	1.61 (1.54–1.69)	44 (24–211)	1.48 (1.03–2.02)
Adjusted## Females	682,780	2,032 (0.30%)	1.46 (0.88–2.10)	2.29 (1.00–3.62)	1.14 (1.11–1.18)	1.55 (1.46–1.64)	31 (18–129)	1.67 (1.01–2.41)

C: Moderate/severe ID								
Model	Number of subjects	Moderate/severe ID (%)	Direct effect^£^ RR (95% CI)	*E*-value (95% CI)	Mediation effect^£^ RR (95% CI)	*E*-value (95% CI)	Proportion mediation^£^ % (95% CI)	Total effect RR (95% CI)
Crude#	1,406,441	1,687 (0.12%)	0.98 (0.48–1.62)	1.14 (1.00–3.59)	1.36 (1.30–1.42)	2.06 (1.92–2.19)	104 (−55–12800)	1.34 (0.65–2.19)
Subgroups								
Adjusted##	1,406,441	1,687 (0.12%)	0.91 (0.46–1.54)	1.43 (1.00–3.77)	1.24 (1.20–1.29)	1.79 (1.69–1.90)	171 (59–1320000)	1.13 (0.58–1.90)
Singletons##	1,365,316	1,628 (0.12%)	0.88 (0.42–1.44)	1.54 (1.00–4.19)	1.29 (1.24–1.35)	1.90 (1.79–2.04)	195 (77–7000)	1.13 (0.55–1.85)
Males##	723,661	984 (0.14%)	0.51 (0.13–1.21)	3.33 (1.00–14.87)	1.27 (1.21–1.34)	1.85 (1.71–2.01)	−39 (−3600–47)	0.65 (0.16–1.49)
Females##	682,780	703 (0.10%)	1.50 (0.60–2.79)	2.37 (1.00–5.02)	1.21 (1.15–1.28)	1.71 (1.57–1.88)	39 (−49–296)	1.82 (0.72–3.33)

Abbreviations: CI, two-sided confidence interval from bootstrapping 1,000 samples; ID, intellectual disability; RR, relative risk from log-binomial regression.

*Note:* # Crude: Outcome and mediation model adjusted for birth year (1998–2002, 2003–2007, 2008–2012, 2013–2015). ## Adjusted: Outcome and mediation model additionally adjusted for maternal age (<20, 20–24, 24–29, 30–34, 34–39, 40–44, >45), paternal age (<20, 20–24, 24–29, 30–34, 34–39, 40–44, >45), maternal psychiatric history at delivery (yes/no), paternal psychiatric history at delivery (yes/no), Maternal and paternal education attainment at delivery (“Grundskola,” “Gymnasium” University), preeclampsia and hypertension. £: Direct effect often referred to as “Controlled Direct Effect” and Mediation usually referred to as “Natural Indirect Effect (NIE).” “Controlled Direct Effect” is the RR of ID comparing offspring to mothers with T1D diagnosis to offspring to mothers without T1D diagnosis, when the preterm covariates are assigned the same value, for example, term. “Natural Indirect Effect” is the RR of ID comparing offspring born preterm to offspring born term assuming all are born to mothers diagnosed with T1D. “Total Effect” is the RR of ID comparing preterm born offspring to mothers with T1D diagnosis to term born offspring to mothers without T1D diagnosis, that is, the RR comparing assumed highest risk group to lowest risk group.

**Table 5.** Hazard ratio for ID by severity

Population	Model		Mild ID		Moderate/severe ID	
		Number of subjects	Rate (cases; person years)	Hazard ratio (95% CI)	Rate (cases; person years)	Hazard ratio (95% CI)
All births	Crude	1,406,441	38.6 (5,229; 3,553,117)	2.04 (1.56–2.67)	12.4 (1,687; 13,553,104)	1.35 (0.77–2.39)
	Model 1			1.78 (1.36–2.33)		1.26 (0.72–2.23)
	Model 2			1.41 (1.07–1.84)		0.93 (0.53–1.65)
*Subgroup:*	Crude	1,365,316	37.9 (4,988; 13,147,786)	2.13 (1.63–2.79)	12.4 (1,628; 13,147,773)	1.39 (0.79–2.46)
	Model 1			1.85 (1.42–2.43)		1.30 (0.74–2.30)
Singletons	Model 2			1.44 (1.10–1.89)		0.91 (0.51–1.61)

*Note:* Crude: adjusted for birth year using splines. Model 1: Crude + additionally adjusted for parental age by natural splines, parental psychiatric history and education. Model 2: Model 1 + additional adjustment for preterm birth.

### Supplementary analyses

Risks of ID in offspring of mothers with T1D were comparable between the sexes (Supplementary Table S2) in term and preterm born (Supplementary Table S3). The association between T1D and ID was not modified by maternal overweight/obesity (Supplementary Table S4), fetal size (Supplementary Table S5), or maternal HbA1c (Supplementary Table S6). Based on the graphs, the risks of ID increase with advancing maternal age in the offspring of mothers with T1D (Supplementary Figure S2). The main analyses were repeated in offspring with at least 10 years’ follow-up and compared between 1998–2002 and 2003–2007 with similar results (Table 2). Power did not allow sibling analyses for controlling of familial confounding.

## Discussion

This nationwide cohort study of 1.4 million live births provides the first population-based risk estimates of ID by severity in offspring of mothers with T1D and shows moderately increased risks of ID in offspring to mothers with T1D. Almost 50% of the increment in risk was mediated by preterm birth, which was accentuated further in case of more severe ID. Accordingly, the effect of T1D alone was most pronounced in term born and manifested as mild cases of ID. We found no modifying effect of maternal HbA1c on the association between T1D and ID risk.

A few previous studies exist of potential associations between maternal T1D and different estimates of cognitive outcomes in the offspring [31–34], including IQ score. However, none of these studies included clinically ascertained diagnoses of ID as in our study. Two studies found an increased risk of lower IQ [34] or lower scores in a standardized national school test in offspring of mothers with T1D compared to controls [33]. In contrast, no significant differences were found comparing primary school grades [32] or “global cognitive scores” [31] between offspring of mothers with and without T1D.

We are aware of only two previous studies investigating the association between maternal T1D and risk of ID in the offspring: one from Sweden and one from Taiwan [35, 36]. The overall risk of ID in offspring of mothers with T1D reported in the Swedish study was comparable to ours [35], whereas risk estimates were higher in the Taiwanese cohort [36]. Differences in results may reflect a higher rate of preterm birth in the Taiwanese study (31.7%), differences in case ascertainment in different health systems, and/or differences in genetic and environmental exposures between countries.

Not all studies have been able to differentiate T1D from type 2 diabetes. A recent population-based study from Sweden covering the years 2004–2008, reported the risks of ID in offspring of mothers with pregestational diabetes (i.e., type 1 or type 2 diabetes, combined) and quantified the mediating effect of preterm birth on risks. The total effect from pregestational diabetes on ID risk was comparable to our risk estimates but with a lower proportion of mediation from preterm birth (17.7% compared to 49% in the current study) potentially due to the mix of both type-1 and type-2 diabetes in the previous study [37]. Even though, these mediating effects were based on similar multivariate models, there were some differences. In our study, not only maternal age and history of psychiatric disease was taken into account, but also the corresponding paternal data.

Results of the current study expand on previous knowledge by presenting risks of different severity of ID, amount of mediation via preterm birth on the risk of ID, and analyses exploring the potential impact of maternal HbA1c on risks. The prospectively collected sample of 1.4 million births with essentially complete follow-up through national health registries, minimized risk of selection bias, enabled adjustment for several important confounders, and investigation of the mediating role of preterm birth. Importantly, diagnoses of ID were not only based on estimates of IQ but also on clinical assessment by specialists that includes an evaluation of adaptive functioning.

We could not perform a more detailed analysis of the association between T1D and subtypes of ID since these are not always reported with ICD10 codes and the individual IQ score is not included in the registries. As in any observational study, our results (HRs) may be due to unmeasured confounding. To address this, we calculated the *E*-values. An *E*-value quantifies the HR that an unmeasured confounder would need to have, with both the exposure and the outcome, conditional on the measured covariates, to fully explain away a specific exposure-outcome association [25]. In our mediation analysis from preterm birth on any ID, the *E*-values were estimated at 1.8 for the direct effect and 1.7 for the mediation effect. For mild ID, the corresponding *E*-values were 2.0 and 1.6. Thus, based on observed estimates of association between our study covariates and ID (HR = 1.5 for T1D and HR = 1.7 for maternal psychiatric history), our results do not provide irrefutable evidence that T1D causes ID together with mediation by preterm birth.

We found that preterm birth was a major driver of the risk of more severe cases of ID. Women with T1D are four to five times more likely to deliver preterm than women without T1D [11]. Risks of preterm birth increase with higher HbA1c levels [38] and maternal obesity [39]. Hyperglycemia during fetal life may lead to permanent changes in neuronal networks [40] and increases the risk of malformations in the central nervous system [41]. Obesity increases inflammation and oxidative stress with possible adverse impact on fetal development [42]. However, we found no modifying effect of maternal HbA1c or obesity on the association between T1D and ID risk.

Higher maternal age increases the risk of offspring ID [8]. In offspring of mothers with T1D, risks of ID were increased also in offspring of younger mothers possibly reflecting accelerated biological age in T1D [29].

## Supporting information

Persson et al. supplementary material Persson et al. supplementary material

## Supplementary material

The supplementary material for this article can be found at https://doi.org/10.1192/j.eurpsy.2024.4.
