# Supplementary material for: Maternal type 1 diabetes, preterm birth, and risk of intellectual disability in the offspring: A nation-wide study in Sweden
Source: Eur Psychiatry. 2024 Jan 22;67(1):e11. doi: 10.1192/j.eurpsy.2024.4 (PMC10964269; doi:10.1192/j.eurpsy.2024.4)
Supplement: Persson et al. supplementary material [file S092493382400004Xsup001.pdf]

SUPPLEMENTARY ONLINE APPENDIX - Tables and figures

List of contents

Table S2 Hazard ratios of ID in subgroups of boys and girls..3

Table S3 Hazard ratios of ID in subgroups by sex and preterm/term births...4

Table S4 Hazard ratios for ID in offspring of mothers with T1D in subgroups by maternal BMI...........................5

Table S5 Hazard ratios for ID in offspring of mothers with T1D in subgroups of size for gestational age...........................6

Table S6 Hazard ratios for ID in offspring of mothers with T1D in subgroups of HbA1c quintiles 7

Figure S1 Cumulative risk of ID in offspring of mothers with T1D compared with offspring of mothers without T1D...8

Figure S2 Hazard ratios for ID by maternal age for offspring of women with and without type-1 diabetes (T1D)…9

SUPPLEMENTARY ONLINE APPENDIX - Tables and figures

Table S1. Diagnostic codes

Diagnosis	ICD-10 code
Intellectual disability	F70 Mild intellectual disabilities<br>F71 Moderate intellectual disabilities<br>F72 Severe intellectual disabilities<br>F73 Profound intellectual disabilities<br>F78 Other intellectual disabilities<br>F79 Unspecified intellectual disabilities
Type 1 diabetes	E 10

ICD: International Classification of Diseases;

Table S2. Hazard ratios for ID in subgroups of boys and girls

Model	Boys			Girls		
	**Number of subjects**	**Rate (Cases; Person years)**	**Hazard ratio (95% CI)**	**Number of subjects**	**Rate (Cases; Person years)**	**Hazard ratio (95% CI)**
Crude	723,661	74 (5,172; 6,970,589)	1.69 (1.26 – 2.26)	682,780	51 (3,351; 6,582,630)	2.24 (1.63 – 3.07)
Adjusted#			1.52 (1.13 – 2.04)			2.00 (1.46 – 2.75)

adjusted for birth year and parental age by natural spline and parental psychiatric history and education. CI: Two-sided 95% confidence interval; Rate: Cases per 100,000 person years

Table S3. Relative risk (Hazard ratios) of ID in offspring to mothers with T1D compared to mother without T1D diagnosis – by sex and preterms/term births.

Population	Preterm born children			Term born children		
	**Number of subjects**	**Rate (Cases; Person years)**	**Hazard ratio (95% CI)**	**Number of subjects**	**Rate (Cases; Person years)**	**Hazard ratio (95% CI)**
Male offspring, adjusted	43,923	189 (806; 426,182)	1.09 (0.70 – 1.70)	679,738	67 (4,366; 6,544,407)	1.28 (0.86 – 1.89)
Female offspring, adjusted	37,949	136 (506; 370,792)	0.75 (0.39 – 1.46)	644,831	46 (2,845; 6,211,837)	2.26 (1.58 – 3.24)

Note: Adjusted models, adjusted for birth year and parental age by natural spline and parental psychiatric history and education ; CI: Two-sided 95% confidence interval; Rate: Cases per 100,000 person years

Table S4. Hazard ratios for ID in offspring of mothers with T1D in subgroups by maternal BMI

Maternal BMI	Number of subjects	Rate (Cases; Person years)	Crude hazard ratio (95% CI)	Adjusted hazard ratio (95% CI)
Normal weight BMI -25	815,764	51 (3,994; 7,769,650)	1.69 (1.16 – 2.47)	1.55 (1.06 – 2.26)
Overweight BMI 25-30	307,938	68 (1,974; 2,917,424)	1.60 (1.05 – 2.44)	1.52 (1.00 – 2.31)
Obesity BMI 30-	145,241	116 (1,514; 1,310585)	1.83 (1.19 – 2.82)	1.59 (1.03 – 2.45)

Model adjusted for fetal sex, parental age and psychiatric history; CI: Two-sided 95% confidence interval; Rate: Cases per 100,000 person years. BMI: Body Mass Index

Table S5. Hazard ratios for ID in offspring of mothers with T1D in subgroups of size for gestational age

**Size for gestational age**	**Number of subjects**	**Rate (Cases; Person years)**	**Crude hazard ratio (95% CI)**	**Adjusted hazard ratio (95% CI)**
Small for gestational age	25,881	253 (629; 249,030)	1.73 (0.65 – 4.64)	1.65 (0.62 – 4.42)
Appropriate for gestational age	1,282,879	58 (7,105; 12,335,667)	2.28 (1.75 – 2.97)	2.04 (1.57 – 2.65)
Large for gestational age	53,768	68 (360; 529,303)	1.49 (0.98 – 2.25)	1.35 (0.89 – 2.05)

#Model adjusted for birth year and parental age by natural splines and parental psychiatric history and education.; CI: Two-sided 95% confidence interval; Rate: Cases per 100,000 person years.

Table S6. Hazard ratios for ID in offspring of mothers with T1D in subgroups of HbA1c quintiles

Quintile	HbA1c (mmol/ml)	Number of children	ID Children (%)	Preterm Children (%)	Maternal age	Compar-ison	Crude Hazard Ratio (95% CI)	Adjusted Hazard Ratio (95% CI)
**Q1**	27 – 49	926	8 (0.86%)	132 (14.3%)	31.6	Reference	-	-
**Q2**	49 – 55	930	3 (0.32%)	170 (18.3%)	31.4	Q2 vs Q1	0.40 (0.11 – 1.52)	0.36 (0.09 – 1.35)
**Q3**	55 – 61	973	6 (0.62%)	210 (21.6%)	31.4	Q3 vs Q1	0.72 (0.25 – 2.07)	0.58 (0.20 – 1.68)
**Q4**	61 – 70	1048	9 (0.86%)	293 (28.0%)	30.8	Q4 vs Q1	1.05 (0.41 – 2.73)	0.80 (0.30 – 2.12)
**Q5**	70 - 177	978	5 (0.51%)	354 (36.2%)	29.5	Q5 vs Q1	0.65 (0.21 – 1.99)	0.44 (0.14 – 1.42)

Model adjusted for birth year and parental age by natural splines and parental psychiatric history and education.; CI: Two-sided 95% confidence interval; HbA1c data categorized into quintiles Q1: 0 to 20% of HbA1c, Q2: 20 to 40% of HbA1c, Q1: 40 to 60% of HbA1c, Q1: 60 to 80% of HbA1c, Q1: 80 to 100% of observed maternal HbA1c.
mmol: micro mol; ml: milli Liter; ID: Intellectual disability; T1D: Type-1 diabetes.

Figure S1. Inverse Kaplan-Meier Survival curves estimating age-cumulative probability of ID in offspring to mothers with T1D, compared to offspring to mothers without T1D

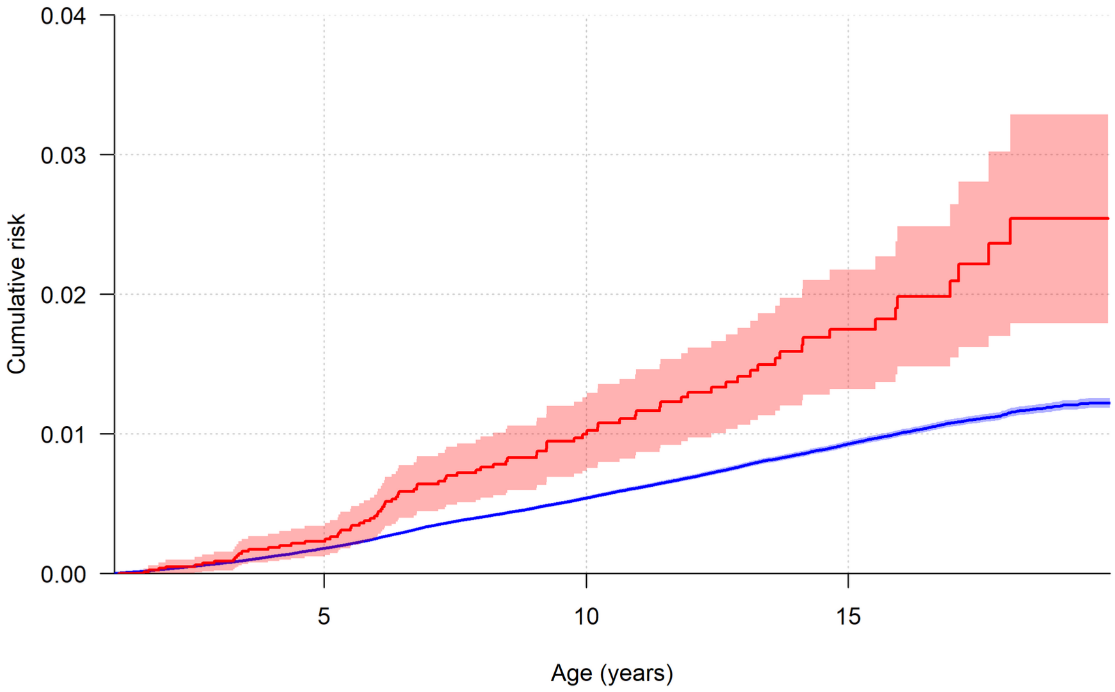

Note: Point estimates and two-sided 95% point-wise confidence intervals. T1D: type-1 diabetes; ID: Intellectual Disability

Figure S2. Risk of ID by maternal age for offspring of women with and without type-1 diabetes (T1D)

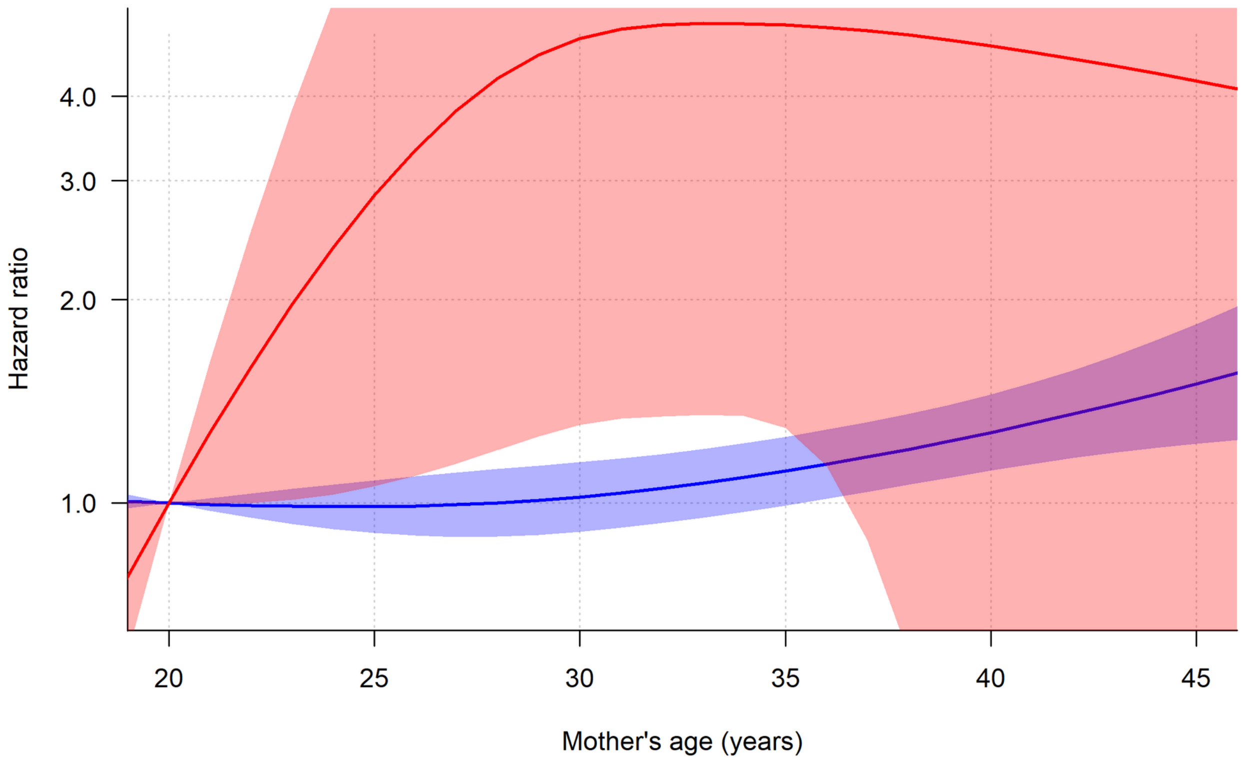

Note: Point estimates and two-sided 95% point-wise confidence intervals. T1D: type-1 diabetes; ID: Intellectual Disability
